# Spatial and temporal variations in environmental variables in relation to phytoplankton composition and biomass in coral reef areas around Unguja, Zanzibar, Tanzania

**DOI:** 10.1186/s40064-015-1439-z

**Published:** 2015-10-28

**Authors:** S. M. Limbu, M. S. Kyewalyanga

**Affiliations:** Department of Aquatic Sciences and Fisheries, University of Dar es Salaam, P.O. Box 35064, Dar es Salaam, Tanzania; Department of Biology, School of Life Sciences, Laboratory of Aquaculture Nutrition and Environmental Health, East China Normal University, 500 Dong Chuan Road, Shanghai, 200241 China; Institute of Marine Sciences, University of Dar es Salaam, Mizingani Road, P.O. Box 668, Zanzibar, Tanzania

**Keywords:** Unguja Island, Phytoplankton, Chlorophyll *a*, Water quality variables, Nutrients

## Abstract

Phytoplankton can indirectly indicate health status of coral reefs due to their sensitivity to changes in water quality parameters. This study explored the spatial and temporal variability in water quality and nutrients in relation to phytoplankton community composition and chlorophyll *a* concentration at Bawe, Mnemba, Chumbe and Pongwe coral reef sites in Unguja Island. In situ measurements of dissolved oxygen, temperature, salinity and pH were done every month for 1 year. Surface water samples were collected for determination of phytoplankton composition, nutrients and chlorophyll *a* concentration. Dissolved oxygen, temperature, salinity and pH did not differ significantly among the four sites (p > 0.05) but showed significant temporal variations among months (p < 0.05). Bawe had significantly higher phosphate concentration (1.45 ± 0.57 µg L^−1^) than Chumbe (0.74 ± 0.53 µg L^−1^), Mnemba (0.42 ± 0.30 µg L^−1^) and Pongwe (0.28 ± 0.10 µg L^−1^; p < 0.05). Similarly, Bawe had significantly higher nitrate concentration (0.81 ± 0.43 µg L^−1^) than Mnemba (0.33 ± 0.14 µg L^−1^) and Pongwe (0.24 ± 0.13 µg L^−1^; p < 0.05) but similar to Chumbe (0.90 ± 0.35 µg L^−1^; p > 0.05). However, values obtained at all the studied sites were less than 3 and 14 mg L^−1^ for phosphate and nitrate, respectively, for eutrophic oceans. Phytoplankton species were dominated by Bacillariophyceae (70.83 %) and some species identified such as *Ceratium* sp., *Dinophysis* sp., *Protoperidinium* sp., *Prorocentrum* sp., *Oscillatoria* sp. and *Dictyocha fibula* are known to produce toxins that affect fish species. Bawe had significantly higher chlorophyll *a* concentration (0.47 ± 0.07 mg L^−1^) than Mnemba (0.33 ± 0.04 mg L^−1^) and Chumbe (0.33 ± 0.04 mg L^−1^; p < 0.05). Chlorophyll *a* concentration was spatially inversely related to distance from Unguja town (p < 0.05) while it was temporally significantly positively correlated with dissolved oxygen, nitrate and phosphate (p < 0.05). The study revealed that, the coral reef sites have low nutrient levels and are in good health. The existence of toxic phytoplankton species suggests careful consumption of fisheries resources at the four coral reef sites and frequent monitoring for Harmful Algal Blooms (HABs) is required. The higher nutrients and chlorophyll *a* concentrations at Bawe Island compared to other sites calls for mechanisms to limit the release of domestic sewage from households and hotels to safeguard the coral reefs.

## Background

Phytoplankton play an important role in the marine food web, biogeochemical cycles and climatic processes (Alvain et al. 2008; Nassar et al. 2014). They initiate the marine food chain, by serving as food to primary consumers such as zooplankton, which in turn transfer energy when consumed by higher trophic animals such as finfish (Saravanakumar et al. 2008). Moreover phytoplankton composition and abundance are considered as natural bio-indicators of water quality variations because of their sensitivity and rapid responses to changes in environmental conditions such as pH, light, temperature, salinity, turbidity and nutrients (Ekwu and Sikoki 2006; Panda et al. 2012; Stanca et al. 2013). Thus, the species composition, relative abundance, spatial and temporal distribution of these aquatic biota are an expression of the environmental health or biological integrity of a particular water body.

Indirectly, phytoplankton can be used to provide indication of the status of coral reefs due to their sensitivity to changes in water quality parameters (Tchernov et al. 2004; Panda et al. 2012). Coral reefs are among the most productive and biologically diverse ecosystems on Earth providing almost a third of the world’s marine fish species and around 10 % of the fish consumed by humans (Moberg and Folke 1999; Stanca et al. 2013). Unfortunately, many coral reefs are in serious decline (De’ath et al. 2012), including those of Zanzibar particularly Unguja Island. The coral reefs in Unguja Island are situated near poor human settlement. They are affected by deforestation, intensive agriculture run off, urbanization, destructive fishing methods, uncontrolled tourism and pollution (McClanahan et al. 2007; Sachithanandam et al. 2013). Unguja’s population is chronically poor and the Island’s 1.5 million residents are heavily dependent on vulnerable marine resources which underpins economic activities accounting for 30 % of GDP (Lange and Jiddawi 2009; Suckall et al. 2014). The release of inorganic nutrients into ocean from domestic sewage has been a major threat to many coral reefs in the area (Bjork et al. 1995). Tourism in the area has grown rapidly, increasing the demand for goods and services in already degraded shallow coral reefs surrounding the Zanzibar coast (Gössling 2001).

Nutrient over-enrichment is considered a major cause of degradation of coral reefs because it leads to a shift from low algal cover to high abundance of fleshy algae (Szmant 2002). Previous studies on coral reefs in the area have reported increased degradation of coral reefs due to growing loads of nutrients, sediments and pollutants discharged from land (Mohammed 2002; Moynihan et al. 2012). Nutrient increase has been shown to cause decreased light penetration, increased sedimentation of organic particles and rapid growth of opportunistic macroalgae (Bjork et al. 1995; Nzali et al. 1998) consequently leading to lower growth of coral reefs. In their study, Bjork et al. (1995) related the decline of the coralline algae they observed to the outlets of sewage water from Unguja town.

Despite the important services provided by corals and the alarming rate on their decline, little documented baseline data exists on algal communities, which are essential tools in assessing the biological integrity of the coral reefs. Fertility and healthiness of coral reefs are reflected through productivity of phytoplankton (Saravanakumar et al. 2008). However studies on phytoplankton abundance and distribution in the coral reefs of Zanzibar is lacking. The lack of historical data on phytoplankton and water quality parameters have been shown to limit attempts to resolve the true impacts on the coral reefs (Johnstone et al. 1998). Much work is still needed to unravel phytoplankton composition and patterns in many remote marine areas that remain largely unexplored, such as those of Unguja coral reefs.

The present study was carried out to analyze the spatial and seasonal variability in water quality variables relative to phytoplankton community structure, diversity and biomass (as chlorophyll *a* concentration). The results of phytoplankton and nutrients were expected to indirectly provide insights on the health condition of coral reefs in Unguja Island. The study hypothesized that the four coral reef sites have different water quality parameters and phytoplankton community structure, diversity and biomass due to their different locations thus experience varying levels of exposure to the monsoon winds and water currents, as well as anthropogenic pressure.

## Methods

### Study sites

The study was conducted at four coral reefs sites in Unguja Island, Zanzibar. Zanzibar Island is in a tropical climate zone characterized by two rainy seasons; a long rainy season between March and May and a short rainy season from October to November (Lugomela et al. 2001). The area receives an average annual rainfall of between 1100 and 1500 mm (Lugomela et al. 2001). The tidal current at the area are semi-diurnal in nature. The Island is also under the influence of monsoon winds and circulation that affect the distribution of nutrients and marine organisms as well as biological processes (Richmond 2011; Peter 2013; Ezekiel 2014).

**Fig. 1:**
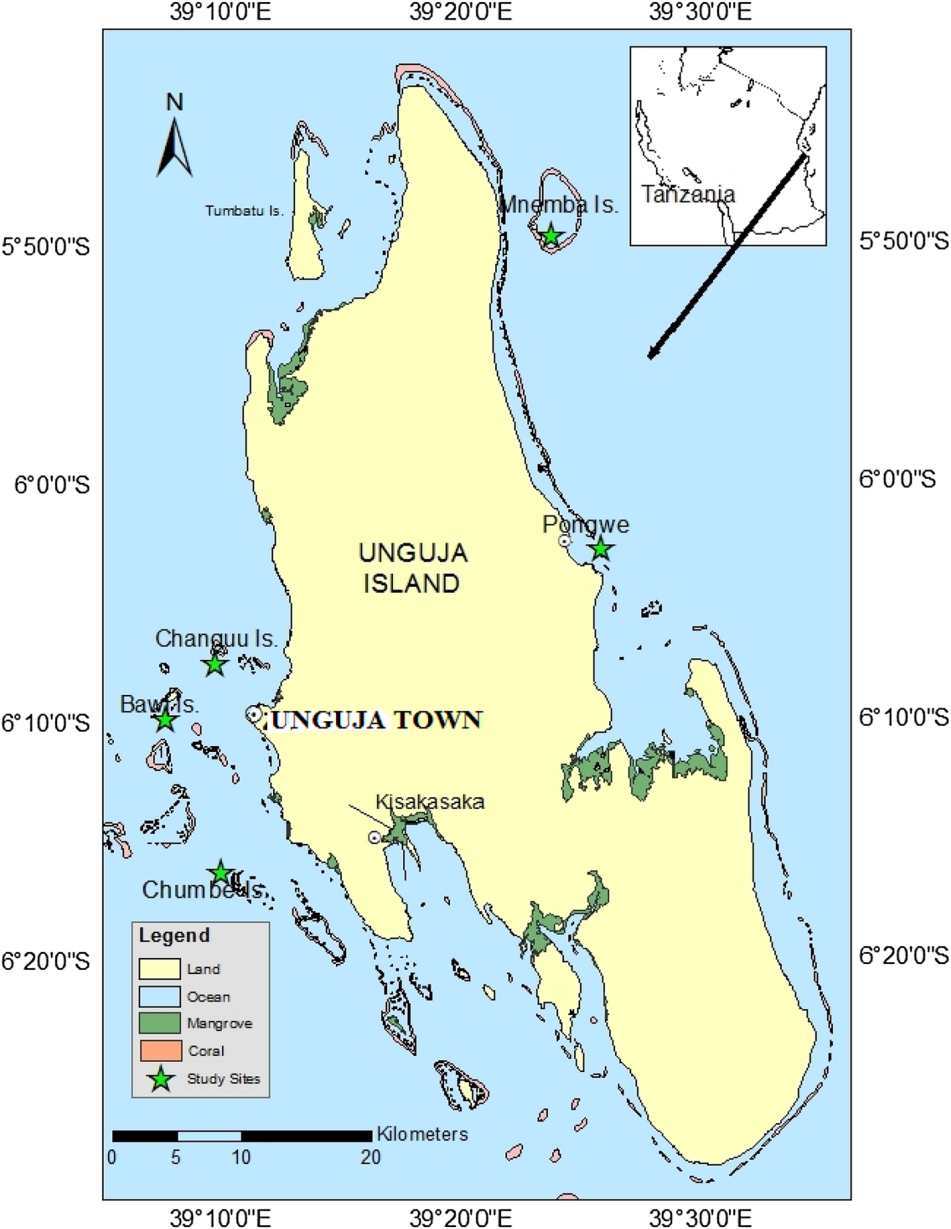
Map of Unguja Island showing the study sites

Four coral reef sites were selected and used during this study to represent sites which are affected by domestic waste disposal, protected areas and those influenced by tourist activities (Fig. 1). The two sampling sites: Bawe-Changuu Island and Chumbe are located on the west coast of Unguja Island whereas the other two sites: Pongwe and Mnemba Island are located on the east coast of Unguja Island. The west coast of Unguja Island is more protected, without prevailing winds and strong currents, while the east coast have high energy coast line with strong winds, currents and tides (Johnstone et al. 1998). All the sites are shallow with depths ranges between 0.50 and 3.00 m at low tide.

Bawe-Changuu Island sites are located at latitudes 6°08′43″S and longitudes 39°08′10″E and 6°07′28″S and 39°98′09″E, 6.33 and 4.80 km, respectively west of Unguja town (Bergman et al. 2000). These reefs are recipients of the town sewage, the disposals and discharge from the harbor activities (Mbije et al. 2002). They are both surrounded by coral reefs and are popular snorkel and dive sites for tourists. During analysis, the data from these two sites were pooled due to their proximity and are herein referred to as Bawe. Chumbe Island (06°16′31″S, 39°10′29″E) is a Marine Protected Area (MPA), located 13.20 km southwest of Unguja town (Bergman et al. 2000). The reef is very lush and well managed with high diversity of hard corals (Mbije et al. 2002). Mnemba Island (5°48′52″ S, 39°23′03″ E) is located about 53.00 km away from Unguja town and has been protected from extraction of resources since 1989 (Bronstein and Loya 2014). Mnemba Atoll has been reported to have the highest species diversity among the reefs in the northern part of Unguja (Masalu 2000). Pongwe (06°01′52″S; 39°25′13″ E) located about 33.00 km form Unguja town is the reef-flats of Zanzibar’s eastern fringing reefs, the site is under heavy utilization from tourists, fishermen and divers (Bronstein and Loya 2014), which increases nutrients levels.

### Sampling procedure

The spatial variation data for measured parameters were obtained at each site as described in the next sections. Data for temporal variation was obtained by averaging the parameter values measured from all the four sampling sites in a month to get a representative value for that particular month for further analysis.

#### Measurement of water quality parameters

At each site, in situ monthly sampling of dissolved oxygen, pH and temperature measurements were done using a Hanna hand-held pH meter (Hanna Instrument: HI 8014-UK). Salinity was measured by using a refractometer (ATAGO S/MILL-Japan). For each parameter, three measurements were taken and their mean value calculated to represent a datum for each sampling occasion for the entire sampling period of 12 months.

#### Determination of nutrients concentrations

At each site, triplicate surface water samples were collected on monthly basis using a water sampler and transported to the laboratory at the Institute of Marine Sciences (IMS) of the University of Dar es Salaam for filtering using 0.25 μm Whatman^®^ GF/F filters. Analysis of phosphate and nitrate in the extracts were determined using a SHIMADZU Spectrophotometer (UV-1201-Japan) as explained in Parsons et al. (1984).

#### Estimation of phytoplankton abundance and diversity

Phytoplankton samples were collected by towing a plankton net (45 μ mesh size; diameter 26 cm) against the currents at subsurface level for 20–30 min. The samples were instantly preserved by fixing with 10 % Lugol’s solution and transported to IMS laboratory for phytoplankton identification. In the laboratory, identification was done using a light microscope equipped with tracing and measuring devices. Identification and classification of phytoplankton were carried out with the aid of standard monographs and publications including Round et al. (1990) and Tomas (1997). The frequency of appearance for a certain phytoplankton species or genus in a sample was used to indicate its abundance. The Shannon–Weaver diversity index (Shannon and Weaver 1949) and Shannon equitability of phytoplankton species among the four sites were estimated using species diversity and richness program developed by Henderson and Seaby (2001) using the following formulae:

1. ${\text{Shannon}}{-}{\text{Weaver diversity index (H}}^{\prime})\, = \, - \sum\limits_{{{\text{i }} = { 1}}}^{\text{s}} {{\text{Pi }}\ell {\text{n(Pi)}}}$where the pi’s are the proportion of all observations in the *i*th species category and S is the total number of species.
2. ${\text{Shannon}}\;{\text{equitability (E}}_{\text{H}} )\, = \,\frac{{\text{H}}^{\prime}}{{{\text{H}}^{\prime}_{ \hbox{max} } }}\; = \;\frac{{\text{H}}^{\prime}}{\ell nS}$where H′_max_ = ln S (equitability assumes a value between 0 and 1, where 1 indicates complete evenness).

#### Determination of chlorophyll *a* concentration

At each study site, 4–6 L of surface water samples were collected in triplicate using plastic bottles every month for determination of chlorophyll *a* concentration for a period of 12 months. The water samples were transported to IMS for laboratory analysis. In the laboratory, water samples were filtered through 0.45 μm millipore membrane filters and extracted in 90 % acetone overnight at 4 °C. Chlorophyll *a* concentration was then measured using a SHIMADZU Spectrophotometer (UV-1201-Japan) according to Parsons et al. (1984).

### Statistical analyses

Results are presented as mean ± standard deviation (SD) and data were tested for normality using Shapiro–Wilk test and homogeneity of variance using Levene’s test. Spatially, none of the measured parameters were normally distributed even after log transformation (p < 0.05). Temporary, water temperature only was normally distributed (W = 0.961, p = 0.226) and has homogeneous variance (F = 1.939, p = 0.085). The other measured parameters were not normally distributed even after log transformation (p < 0.05). Thus, temporal water temperature was analyzed using one way analysis of variance (ANOVA) followed by Tukey’s post hoc test for specific differences. Kruskal–Wallis (H) test was utilized to determine spatial significant differences in chlorophyll *a*, dissolved oxygen, water salinity and pH among the four sites and temporally among the 12 sampling months. When significant differences were detected, pairwise comparisons were conducted using the Mann–Whitney (*U)* test. Results for ANOVA and Kruskal–Wallis tests are reported as F and H values, respectively, with subscripts in F results denoting degrees of freedom (df). Spearman’s correlation was used to show any association among different parameters measured both spatially and temporally during the study. All statistical analyses were performed using SPSS for windows version 20 (SPSS, Inc). Results with p ≤ 0.05 were considered statistically significant for all statistical tests except Spearman’s correlations which used a p ≤ 0.01.

## Results

### Spatial variations

#### Water quality parameters

Dissolved oxygen, temperature, salinity and pH did not differ significantly among the four sites (dissolved oxygen, H = 0.602, df = 3, p = 0.896; temperature, H = 1.058, df = 3, p = 0.787; salinity, H = 0.391, df = 3, p = 0.942 and pH, H = 3.030, df = 3, p = 0.387; Table 1).

**Table 1:** Water quality parameters (mean ± SD) measured during the study period at four coral reef sampling sites

Parameter	Pongwe	Mnemba	Chumbe	Bawe
Temperature (°C)	27.72 ± 1.20	27.49 ± 1.21	27.67 ± 1.40	27.51 ± 1.34
Salinity (ppt)	34.92 ± 1.46	35.00 ± 1.49	34.92 ± 2.13	35.08 ± 1.59
Dissolve oxygen (mg L^−1^)	5.77 ± 0.42	5.80 ± 0.38	5.88 ± 0.54	5.78 ± 0.39
pH	7.91 ± 0.12	7.93 ± 0.11	7.93 ± 0.12	7.96 ± 0.10

#### Nutrients concentrations

**Fig. 2:**
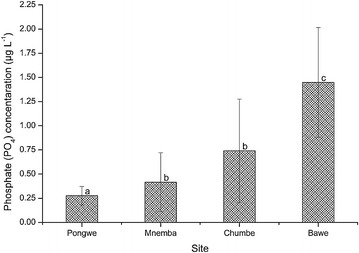
The spatial variation in phosphate (PO_4_) concentration at the four sites during the study period. *Different letters* (*a*, *b* and *c*) above *bars* indicate significant differences (p ≤ 0.05)

The values of phosphate varied between 0.28 ± 0.10 µg L^−1^ and 1.45 ± 0.57 µg L^−1^ recorded at Pongwe and Bawe respectively (Fig. 2). The concentration of phosphate differed significantly among the four sites (H = 39.607, df = 3, p < 0.001). Mann–Whitney’s test showed significantly higher phosphate concentration at Bawe than at Chumbe (*U* = 463.000, p = 0.037), Mnemba (*U* = 232.500, p < 0.001) and Pongwe (*U* = 121.500, p < 0.001). Similarly, the concentration of phosphate recorded at Chumbe was significantly higher than that recorded at Pongwe (*U* = 381.500, p = 0.003). Mnemba had significantly higher phosphate concentration than Pongwe (*U* = 408.500, p = 0.007). However, Mnemba had statistically similar phosphate concentration to Chumbe (*U* = 517.000, p = 0.140). The spatial concentration of phosphate decreased as the sampling site distance increased from Unguja town (r = −0.415, p < 0.001).

**Fig. 3:**
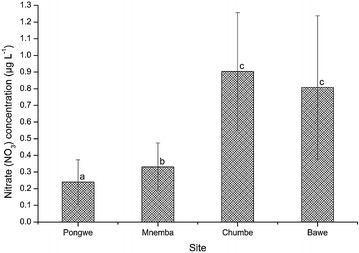
The spatial variations in nitrate (NO_3_) concentration at the four sites during the study period. *Different letters* (*a*, *b* and *c*) above *bars* indicate significant differences (p ≤ 0.05)

Nitrate concentration varied from a minimum value of 0.24 ± 0.13 µg L^−1^ recorded at Pongwe (Fig. 3). The maximum value of nitrate concentration of 0.90 ± 0.35 µg L^−1^ was recorded at Chumbe. The concentration of nitrate differed significantly among the four sampled sites (H = 26.016, df = 3, p < 0.001). Nitrate levels at Bawe were significantly higher than those at Mnemba (*U* = 439.000, p = 0.018) and Pongwe (*U* = 246.500, p < 0.001). Equally, Chumbe had significantly higher nitrate concentration than Mnemba (*U* = 469.500, p = 0.044) and Pongwe (*U* = 314.500, p < 0.001). Mnemba had statistically higher nitrate concentration than Pongwe (*U* = 397.000, p = 0.005). However, Bawe and Chumbe had statistically similar nitrate concentrations (*U* = 641.500, p = 0.942). The spatial concentration of nitrate decreased significantly as the distance of the sampling site increased from Unguja town (r = −0.279, p = 0.001).

#### Phytoplankton composition

**Fig. 4:**
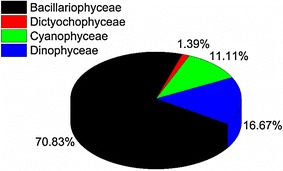
Phytoplankton composition from all four coral reef sites during the entire year of study

A total of 72 phytoplankton species belonging to four families were identified at the four coral reef sites during the entire 12 months of the study period. Bacillariophyceae was the most dominant phytoplankton class with 51 species representing 70.83 % (Fig. 4). The most dominant species of Bacillariophyceae were *Rhizosolenia* sp., *Nitzschia* sp., *Chaetoceros* sp., *Bacteriastrum* sp. and *Navicula* sp. Dinophyceae was the second dominant group with 12 species representing 16.67 %. It was dominated by *Ceratium* sp., *Dinophysis* sp., *Protoperidinium* sp. and *Prorocentrum* sp. Cyanophyceae had 11.11 % represented by *Oscillatoria* sp., *Nostoc* sp., *Schizothrix* sp. and *Johannesbaptistia pellucida*. *Dictyocha fibula* was the only species in the group of Dictyochophyceae (1.39 %) identified at Chumbe.

All the four coral reef sites had identical types of phytoplankton species. The Shannon equitability values were 0.86, 0.84, 0.83 and 0.82 for Chumbe, Pongwe, Mnemba and Bawe, respectively. Chumbe Island recorded relatively the highest number of species (57). The other sites had similar number of phytoplankton species, i.e., 47, 48 and 49 for Pongwe, Bawe and Mnemba, respectively. Furthermore, regardless of the study site, the dominant group was Bacillariophyceae, which had more number of species that occurred throughout the year. The Shannon–Weaver diversity indices were 3.67, 3.60, 3.55 and 3.49 for Chumbe, Pongwe, Mnemba and Bawe, respectively.

#### Chlorophyll *a* concentration

**Fig. 5:**
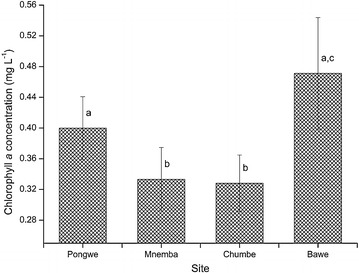
The spatial variations in chlorophyll *a* concentration at the four sites during the study period. *Different letters* (*a*, *b* and *c*) above *bars* indicate significant differences (p ≤ 0.05)

The minimum and maximum mean values of chlorophyll *a* concentrations were 0.33 ± 0.04 and 0.47 ± 0.07 mg L^−1^ recorded at Chumbe and Bawe, respectively (Fig. 5). The spatial variations in chlorophyll *a* concentration was significantly different among the four sampled sites (H = 11.844, df = 3, p = 0.008). Significantly higher chlorophyll *a* concentration was recorded at Bawe than at Mnemba (*U* = 422.000, p = 0.011) and Chumbe (*U* = 384.500, p = 0.003). Pongwe had significant higher chlorophyll *a* concentration than Chumbe (*U* = 463.000, p = 0.037) and marginally with Mnemba (*U* = 477.000, p = 0.054). However, insignificant difference in chlorophyll *a* concentration existed between Bawe and Pongwe (*U* = 602.000, p = 0.604). Mnemba and Chumbe sites showed statistically similar chlorophyll *a* concentrations (*U* = 646.000, p = 0.982). The spatial concentration of chlorophyll *a* decreased significantly as the distance of the sampling site increased from Unguja town (r = −0.245, p = 0.003).

### Temporal variations

#### Water quality parameters

**Fig. 6:**
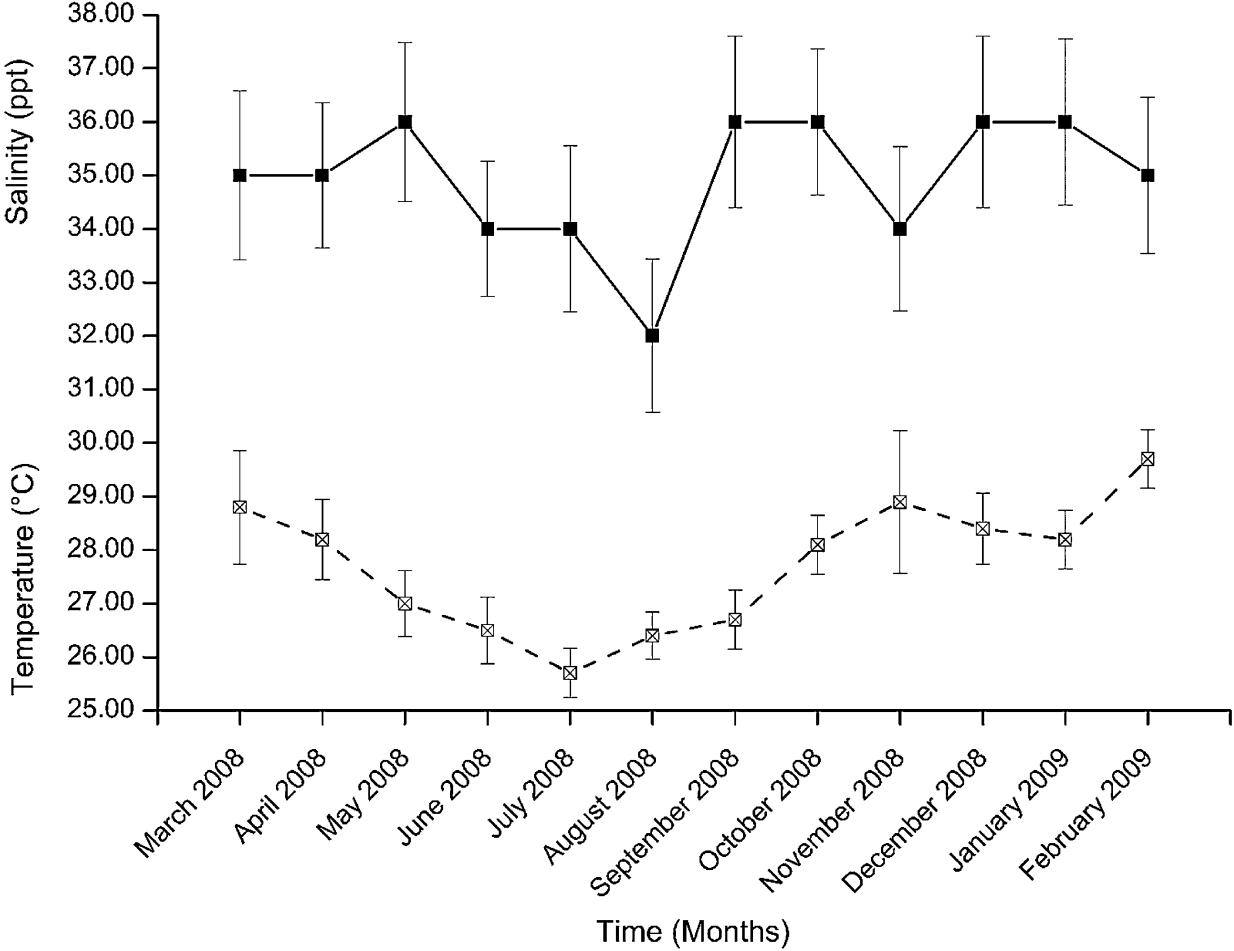
Temporal variation in mean values (± SD) of salinity and water temperature during the study period

Salinity showed heterogeneous temporal variation with a minimum value of 32.00 ± 1.43 ppt recorded in August 2008 (Fig. 6). The maximum value (around 36 ppt) was recorded in 5 months. Salinity was significantly different among the sampling months (H = 21.569, df = 11, p = 0.028). The salinity values in August was significantly lower than that in March, April, May, September, October and December (2008), as well as in January and February (2009) (p < 0.05).

Water temperature showed a regular seasonal cycle (Fig. 6). The maximum water temperature was recorded in February 2009 (29.70 ± 0.55 °C) and the minimum was in July 2008 (25.70 ± 0.46 °C). Water temperature differed significantly among the sampling months (F_11,96_ = 89.545, p < 0.001). February 2009 had significantly the highest water temperature of all the months (p < 0.05). On the other hand, July 2008 had significantly the lowest water temperature of all the months (p < 0.05).

**Fig. 7:**
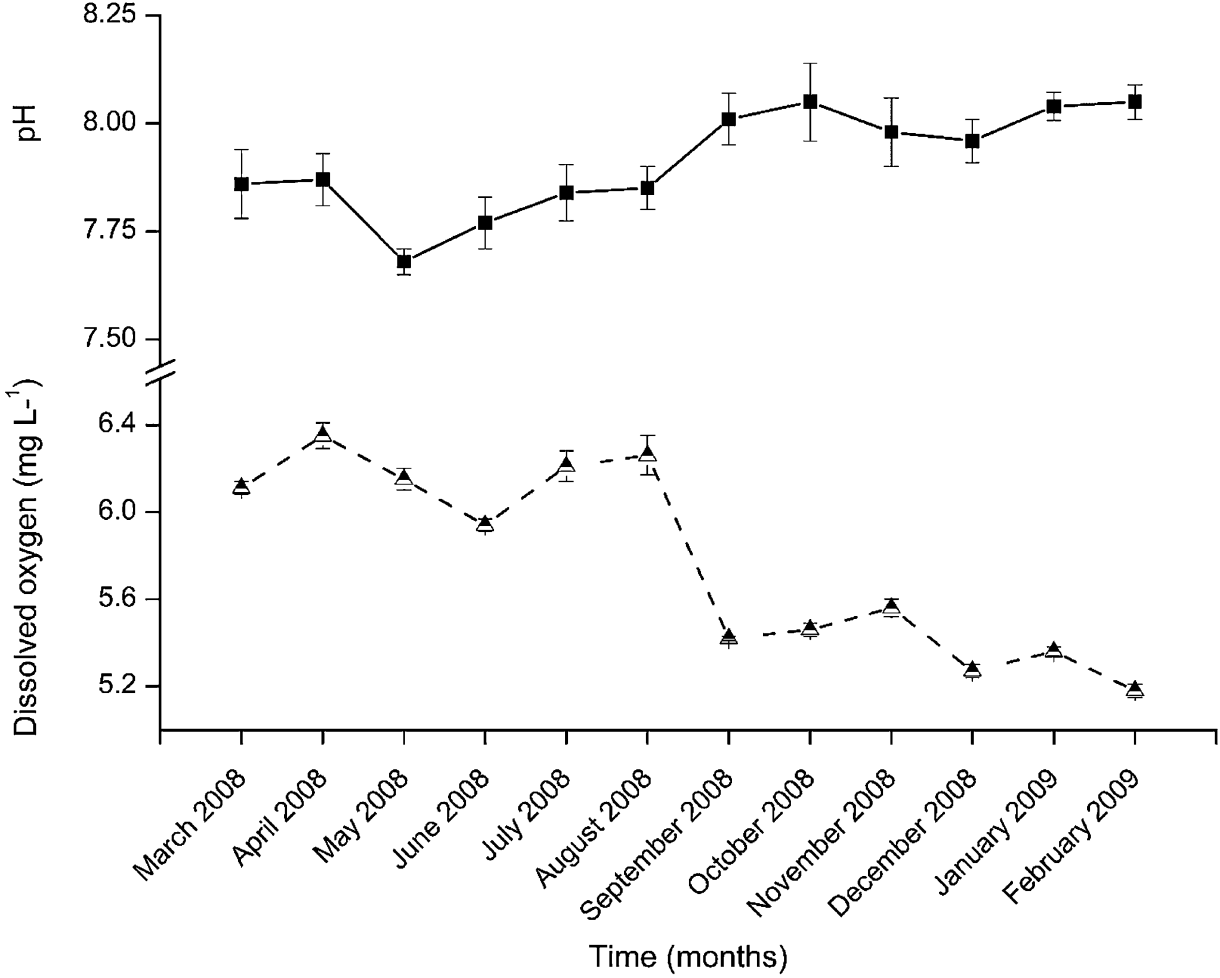
Fluctuation in the mean temporal variations (± SD) in pH and dissolved oxygen during the study

The results of dissolved oxygen and pH are shown in Fig. 7. While dissolved oxygen decreased with time (from August 2008 onwards), pH tended to increase within the same period. Nevertheless, all the dissolved oxygen values were above 5.0 mg L^−1^ and pH values remained alkaline for all months. The highest pH value (8.05 ± 0.04) was obtained in October 2008 and January 2009. The lowest value (7.68 ± 0.07) was recorded in May 2008. The values of pH varied significantly among the sampling months (H = 34.079, df = 11, p < 0.001). The pH values for May, June and September 2008 were significantly lower than the rest of the months (p < 0.05). However, no significant difference in water pH was recorded between March and April, July and August 2008 as well as between February 2009 and October 2008 and January 2009 (p > 0.05). Equally, November and December 2008 had statistically similar values of water pH (p > 0.05). The highest dissolved oxygen (6.35 ± 0.06 mg L^−1^) was observed in April 2008 whereas the lowest (5.18 ± 0.03 mg L^−1^) was recorded in February 2009. Dissolved oxygen differed significantly among the sampling months (H = 34.784, df = 11, p < 0.001). All the measured dissolved oxygen values differed significantly among the sampling months (p < 0.05).

#### Nutrients concentration

**Fig. 8:**
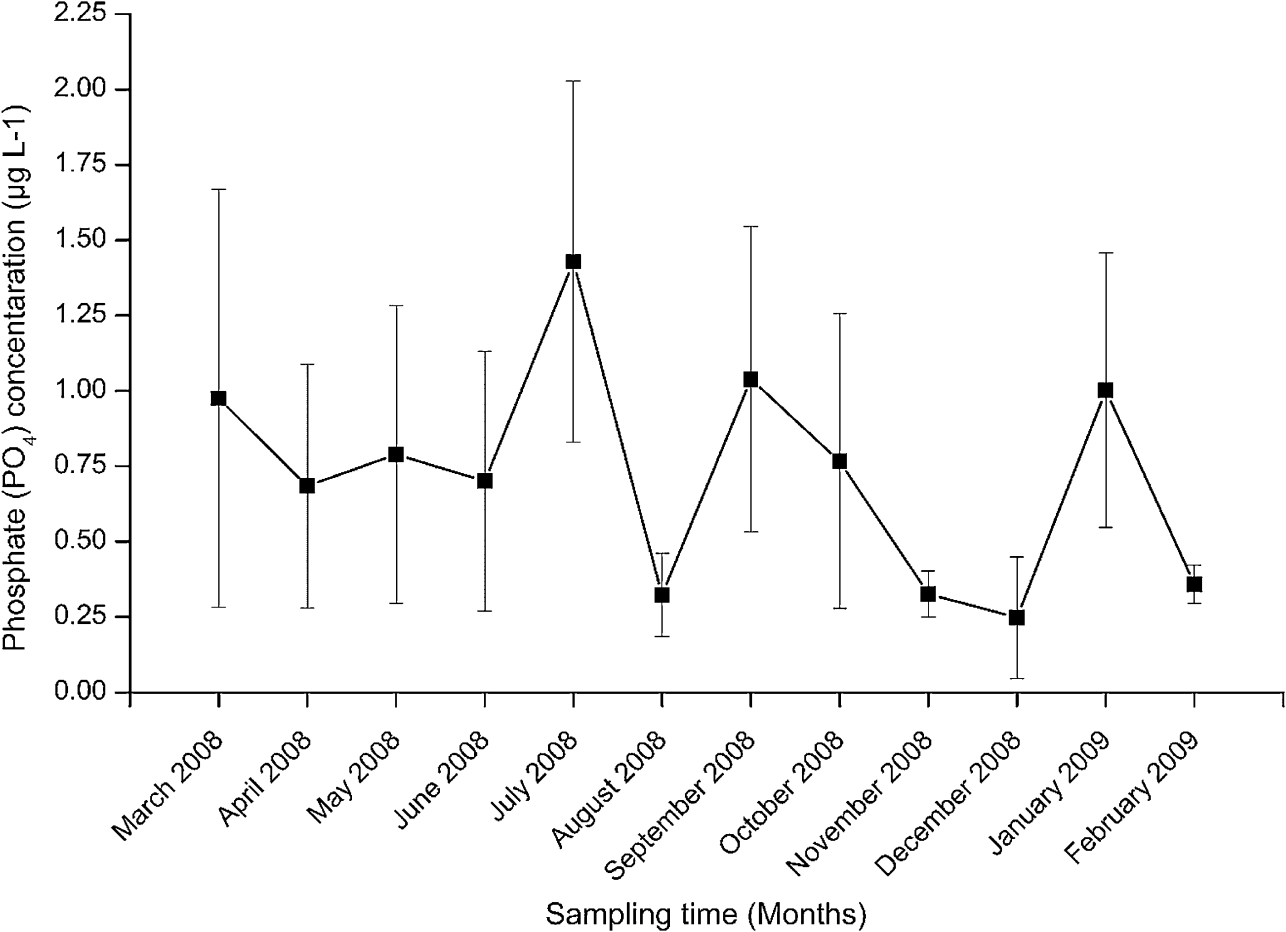
The temporal variations in phosphate (PO_4_) concentration at the four sites during the study period

The values of phosphate concentration varied between 0.25 ± 0.20 and 1.43 ± 0.60 µg L^−1^ recorded in December and July 2008, respectively (Fig. 8). The concentration indicated a significant seasonal trend among the sampling months (H = 23.705, df = 11, p = 0.014). On one hand, the phosphate concentration recorded in July 2008 was significantly higher than that recorded in April, May, June, August, October, November, December 2008 and February 2009 (p < 0.05). Similarly, January 2009 had significantly higher phosphate concentration than August, November, December 2008 and February 2009 (p < 0.05). On the other hand, the phosphate concentration recorded in December 2008 was significantly lower than that recorded in March, April, May, June, September, October, 2008 and January 2009 (p < 0.05). The phosphate concentration recorded in February 2009 was significantly lower than that recorded in March, April, May, September, October and November 2008 (p < 0.05).

**Fig. 9:**
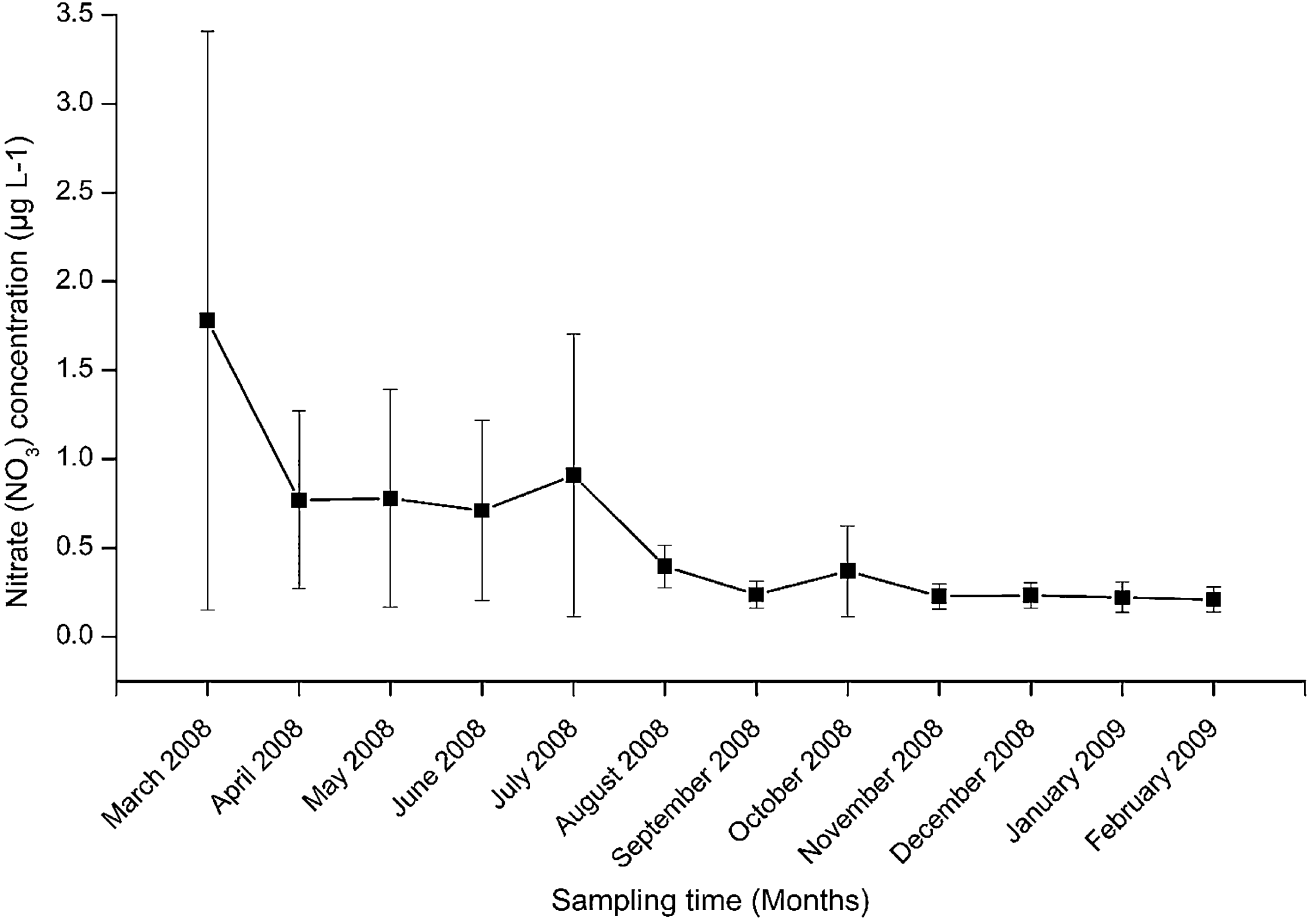
The temporal variations in nitrate (NO_3_) concentration at the four sites during the study period

The values of nitrate concentration varied between 0.21 ± 0.07 and 1.78 ± 1.63 µg L^−1^ recorded in December and March 2008, respectively (Fig. 9). Nitrate concentration indicated significant temporal variation among the different sampling months (H = 48.993, df = 11, p < 0.001). The nitrate concentration recorded in March 2008 was significantly higher than that recorded in November and December 2008 as well as January and February 2009 (p < 0.05). Equally, the nitrate concentrations recorded in the months of April, May and June 2008 were significantly higher than those recorded in August, September, October, November and December 2008 as well as January and February 2009 (p < 0.05).

#### Phytoplankton composition

The temporal distribution of phytoplankton showed that *Rhizosolenia* sp. were abundant in all the sampling months except July 2008. *Oscillatoria* sp. were abundant during the months of March, April, October and December, 2008; and also in January and February 2009. *Nitzschia* sp., *Chaetoceros* sp. and *Bacteriastrum* sp. were abundant in the months from July 2008 to February 2009 and *Ceratium* sp. were abundant in the later months, from September 2008 to February 2009. *Navicula* sp. were abundant in August, September 2008 and January and February 2009. *Dinophysis* sp. were abundant only in December 2008 and February 2009. *Nostoc* sp., were abundant in July and December 2008, January and February 2009. *Protoperidinium* sp. and *Schizothrix* sp. were abundant in September 2008 whereas *Johannesbaptistia pellucida* were abundant only in July 2008.

#### Chlorophyll *a* concentration

**Fig. 10:**
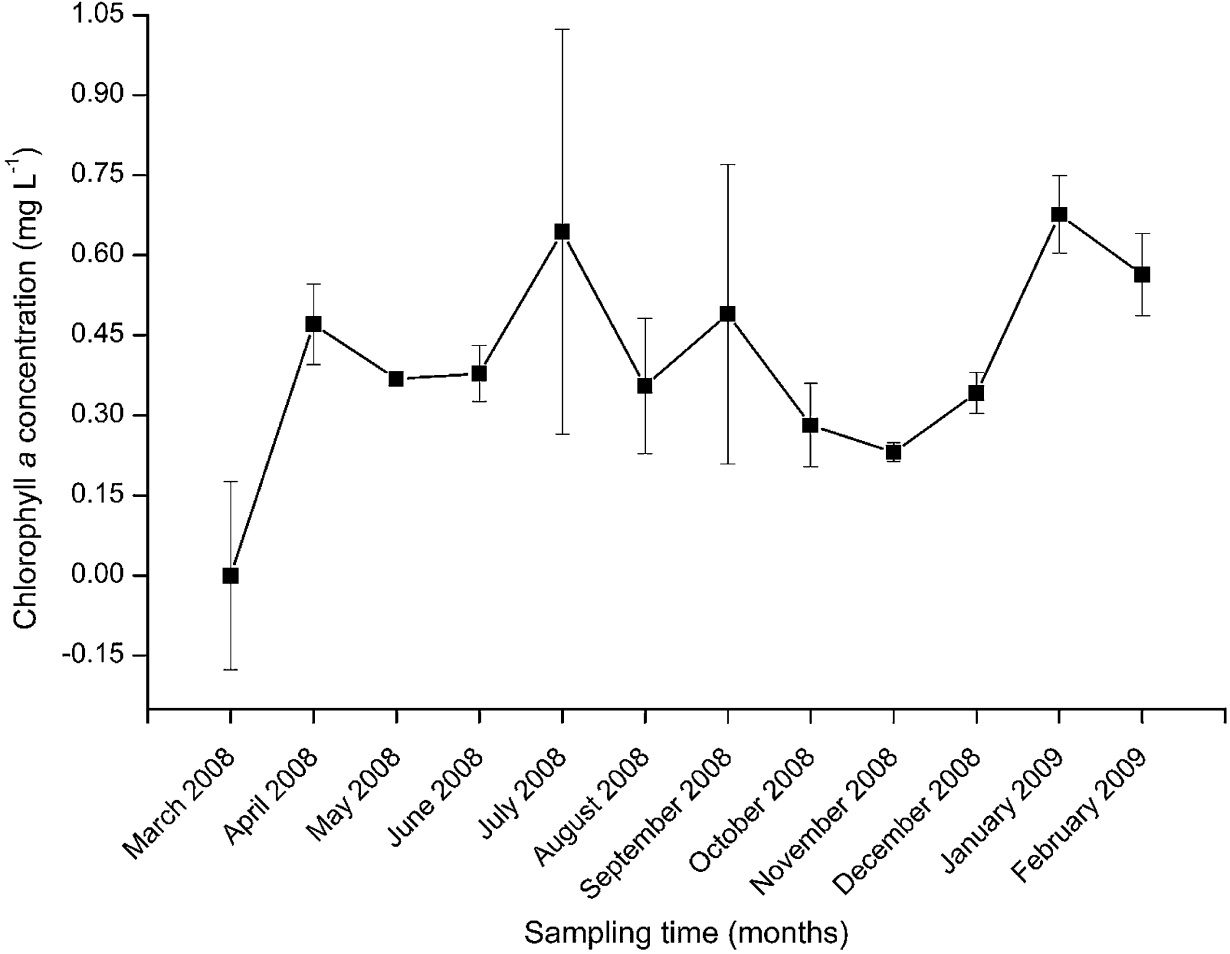
The temporal variations in chlorophyll *a* concentration during the study period

The temporal variation in chlorophyll *a* concentration during the twelve months of sampling period are shown in Fig. 10. The mean chlorophyll *a* concentration was below detection limit during the month of March 2008. There were two peaks in chlorophyll *a* concentration recorded in the months of July 2008 (0.64 ± 0.38 mg L^−1^) and January 2009 (0.68 ± 0.08 mg L^−1^). Sampling months had significant difference in chlorophyll *a* concentration (H = 25.358, df = 11, p = 0.008). The concentration of chlorophyll *a* recorded in March 2008 was significantly lower than that recorded in all other months. The concentration of chlorophyll *a* recorded in April 2008 was significantly higher than that recorded in May, October, November and December 2008 (p < 0.05). Similarly, the months of July 2008 and January 2009 had significantly higher chlorophyll *a* concentration than May, June, August, October, November and December 2008 (p < 0.05). The month of February 2009 also had significantly higher chlorophyll *a* concentration than months of October, November and December 2008 (p < 0.05).

### The relationship between chlorophyll *a* concentration, water quality parameters and nutrients

The results of temporal correlation between chlorophyll *a* concentration and water quality parameters revealed positive and negative influence. Dissolved oxygen had a positive significant correlation with chlorophyll *a* concentration (r = 0.68, p = 0.044). Temperature (r = −0.036, p = 0.667) and salinity (r = −0.011, p = 0.893) had negative insignificant correlation with chlorophyll *a* concentration while pH (r = 0.056, p = 0.507) had positive insignificant correlation.

The concentration of chlorophyll *a* had significant positive correlations with the concentrations of phosphate (r = 0.216, p = 0.009) and nitrate (r = 0.250, p = 0.003). The concentration of phosphate and nitrate also significantly positively correlated (r = 0.710, p < 0.001).

## Discussion

This study aimed to determine the spatial and seasonal variability in water quality variables and nutrients relative to phytoplankton community structure, diversity and biomass in order to indirectly provide insights on the health status of coral reefs in Unguja Island. The nutrient concentration results revealed significant higher phosphate concentration at Bawe than at Chumbe, Mnemba and Pongwe. Similarly, nitrate was also significantly higher at Bawe and Chumbe than at Mnemba and Pongwe. Moreover, results showed that the concentrations of phosphate and nitrate decreased as the sampling site distance increased from Unguja town. These results are in agreement with those obtained by Bjork et al. (1995). This is due to the influence of domestic sewage inflows from Unguja town to Bawe site. Bawe is the closest site from Unguja town, located at about 6.33 km only compared to 13.20 km for Chumbe, 53.00 km for Mnemba and 33.30 km for Pongwe. Due to its proximity to Unguja town, more domestic sewage water from households and hotels in the town released into the ocean find their way to the site thereby increasing the concentration of nutrients.

Despite the higher concentrations of phosphate and nitrate at Bawe, its coral reefs are considered health because the levels of nutrients for unhealthy coral reef ecosystems are approximately 3 mg L^−1^ of P as orthophosphate and organophosphate and 14 mg L^−1^ of N as nitrate (Bell 1992; Goreau and Thacker 1994). These levels are still higher compared to highest concentrations of 1.45 ± 0.23 µg L^−1^ for phosphate and 0.90 ± 0.16 µg L^−1^ for nitrate recorded in the present study. However, release of nutrients in the coral reefs should be limited to avoid excessive growth of phytoplankton and macroalgae, which may affect the growth of coral reefs by competing for light, nutrients and space or even through development of harmful algal blooms (HBAs) in the case of phytoplankton.

The current study showed dominance of phytoplankton by Bacillariophyceae class (70.83 %). Similar results in marine environment have been obtained by Saravanakumar et al. (2008), Saifullah et al. (2014) and Moto (2015) who obtained 78.85, 76.40 and 67.69 % of Bacillariophyceae composition, respectively. The high abundance of Bacillariophyceae is due to their ability to proliferate in the aquatic environment. Bacillariophyceae have been shown to exhibit a boom and bust (or bloom and bust) lifestyle in freshwater and marine environments (Richard and Stickley 2010). They effectively utilize nutrients and light availability to allow them compete and quickly dominate the other phytoplankton species (Buchan et al. 2014). Based on this capability, Furnas (1990) classified them as opportunistic r-strategists (i.e. those organisms whose ecology is defined by a high growth rate, r). This ability make Bacillariophyceae the most abundant and ubiquitous group in terms of ecosystems and water chemistry (Coelho et al. 2013; Desrosiers et al. 2013) making them responsible for approximately 20 % of global aquatic photosynthesis (Rosenwasser et al. 2014). The dominance of Bacillariophyceae in coral reefs as obtained in the present study is likely to contribute to nutrient cycling and gas exchange (Barott et al. 2011) subsequently promoting growth and abundance of corals and algae.

Dinophyceae ranked second in dominance representing 16.67 % dominated by *Ceratium* sp., *Dinophysis* sp., *Protoperidinium* sp. and *Prorocentrum* sp. while Cyanophyceae was the third group (11.11 %) represented by *Oscillatoria* sp., *Nostoc* sp., *Schizothrix* sp. and *Johannesbaptistia pellucida*. These results are similar to those obtained by Bajarias (2000) working in the South China Sea; Dayala et al. (2014) at Cochin estuarine and Moto (2015) in Zanzibar waters in the Indian Ocean. Dinophyceae are known to form symbiotic relationship with coral reefs enabling them to flourish in coral reef sites than other phytoplankton species (Hackett et al. 2004). In this relationship, Dinophyceae symbionts play a significant role in the nutrition of coral reefs and physiology by translocating enough photosynthetically fixed carbon to meet the hosts’ respiratory demands. Meanwhile, they facilitate the assimilation and conservation of nitrogen (Santos et al. 2004) while getting enough carbon for photosynthesis allowing them to multiply and increase in number (Badylak and Phlips 2004). The symbiont diversity has also shown to help coral reefs survive moderate climate change (Baskett et al. 2009).

The general ecological implications of the composition of phytoplankton species obtained at the four sites during the entire 12 months of the study period indicate existence of potentially harmful microalgae. Some of the Dinophyceae, Cyanophyceae and Dictyochophyceae species identified in the present study are known to produce toxins and affect fish species. *Ceratium* sp., *Dinophysis* sp., *Protoperidinium* sp. and *Prorocentrum* sp. were reported by Siyambalapitiya et al. (2012) as potentially harmful red tide forming Dinophyceae. *Protoperidinium* sp. was identified as the causative agent of a group of toxins known as azaspiracids (James et al. 2003; Blanco et al. 2005). High abundance of certain species of Cyanophyceae such as *Oscillatoria* sp. has been shown to produce microcystins toxins (Kyewalyanga and Lugomela 1999) and are associated with aquatic pollution (Ekwu and Sikoki 2006). *Dictyocha fibula,* a Dictyochophyceae species has been shown to be harmful to fish gills at high concentrations (Koutsodendris et al. 2015). Based on the present study, the four sites have low nutrients but bear a problem of existence of toxic phytoplankton species. Although the concentration is somehow low, it is hereby suggested that frequent monitoring programs need to be conducted to ensure that the consumption of seafood from the studied areas does not lead to health problems.

The spatial variations in chlorophyll *a* concentration during the study indicated higher amount at Bawe. The results further showed that, the concentration of chlorophyll *a* was significantly positively correlated with phosphate and nitrate. Higher chlorophyll *a* concentration at Bawe is most likely due to higher concentration of phosphate and nitrate. Studies in oceans have shown that the biomass of phytoplankton (indirectly measured using chlorophyll *a*) is limited by phosphate and nitrate (Tantanasarit et al. 2013; Turner and Rabalais 2013). Consequently, since Bawe had higher levels of both phosphate and nitrate, it resulted in higher concentration of chlorophyll *a*. Most chlorophyll *a* values obtained in the present study are not worrisome because they have not reached the eutrophication threshold value at or below an annual mean of 0.5 mg m^−3^ as suggested by Bell (1992). However, a mean value of 0.47 ± 0.04 mg L^−1^ obtained at Bawe provides a warning signal to reduce the release of un-treated sewage water into the ocean.

The current results showed peak chlorophyll *a* concentration in July 2008 and January 2009 during the long monsoon winds. Similar results of higher chlorophyll *a* concentration during monsoon winds have been obtained by Strutton et al. (2015). Several recent studies in main land Tanzanian and Zanzibar waters (Peter 2013; Ezekiel 2014; Moto 2015) have also revealed higher chlorophyll *a* during the months of July and January due to the influence of southeast monsoon winds. During monsoon season, strong wind mixing brings up nutrient-rich waters from deep waters to the surface, thereby stimulating phytoplankton growth and production.

The current study indicated that, dissolved oxygen had a positive significant correlation with chlorophyll *a* concentration whereas pH, temperature and salinity were all negatively correlated. Negative correlation between chlorophyll *a* concentration and salinity has been reported by Macedo et al. (2001) and Yin (2002), pH by Rahaman et al. (2013) and temperature by Dayala et al. (2014) similar to the results obtained in this study. The positive correlation between chlorophyll *a* concentration and dissolved oxygen is due to necessity of the latter for phytoplankton biogeochemical processes. Dissolved oxygen is one of the most important water quality parameters for phytoplankton health because of its influence in a number of biogeochemical processes such as respiration and metabolism that affect their life (Iriarte et al. 2015). During photosynthesis process, oxygen is released as a byproduct, thus as phytoplankton biomass increases (as determined by chlorophyll *a* concentration), the rate of photosynthesis also increases releasing more oxygen into the water. This phenomenon is beneficial to the coral reefs at the four studied sites.

## Conclusion

The current results indicated dominance of Bacillariophyceae and Dinophyceae both spatially and temporally in coral reefs. These results imply that, at present state phytoplankton are likely to contribute to nutrient cycling and gas exchange for the benefit of both corals and algae. However, release of nutrients in the coral reefs should be limited to avoid excessive growth of Dinophyceae (most of which are potentially harmful microalgae), Bacillariophyceae and possibly macroalgae that may compete with the host symbiotic algae for nutrients, light and inorganic carbon affecting the development of coral reefs. Spatially, the coral reefs sites at the moment have low nutrient levels and are in good health. Temporally, chlorophyll *a* concentration at the four coral reef sites is regulated by the availability of phosphate, nitrate and dissolved oxygen as well as monsoon winds. The existence of toxic phytoplankton species at the four coral reef sites calls for regular monitoring programmes on HABs and careful consumption of the associated seafood. The higher spatial chlorophyll *a* concentration at Bawe compared to other sites demands for mechanisms in Unguja Island to limit the release of domestic sewage from households and hotels to safeguard the coral reefs at the island. The present study provides a baseline guide on the status of coral reefs by using phytoplankton and nutrients for one year. This study should be followed by extensive study covering all the existing coral reefs sites in coastal waters of Tanzania for longer periods of time.
